# Efficient chimeric antigen receptor targeting of a central epitope of CD22

**DOI:** 10.1016/j.jbc.2023.104883

**Published:** 2023-06-01

**Authors:** Nicholas Paul Casey, Clara Helena Klee, Anne Fåne, Benjamin Caulier, Agnieszka Graczyk-Jarzynka, Marta Krawczyk, Klaudyna Fidyt, Sarah E. Josefsson, Hakan Köksal, Pierre Dillard, Elzbieta Patkowska, Malgorzata Firczuk, Erlend B. Smeland, Magdalena Winiarska, June H. Myklebust, Else Marit Inderberg, Sébastien Wälchli

**Affiliations:** 1 Translational Research Unit, Section of Cellular Therapy, Department of Oncology, Oslo University Hospital, Oslo, Norway; 2 Faculty of Medicine, Center for Cancer Cell Reprogramming (CanCell), Institute for Clinical Medicine, University of Oslo, Oslo, Norway; 3 Department of Molecular Cell Biology, Institute for Cancer Research, Oslo University Hospital, Oslo, Norway; 4 Department of Immunology, Medical University of Warsaw, Warsaw, Poland; 5 Laboratory of Immunology, Mossakowski Medical Research Institute, Polish Academy of Sciences, Warsaw, Poland; 6 Department of Cancer Immunology, Institute for Cancer Research, Oslo University Hospital, Oslo, Norway

**Keywords:** antibody, B-cell lymphoma, bispecific T-cell engager, cancer therapy, chimeric antigen receptor, immunotherapy, *in vivo* imaging

## Abstract

Chimeric antigen receptor (CAR) T-cell therapy has had considerable success in the treatment of B-cell malignancies. Targeting the B-lineage marker CD19 has brought great advances to the treatment of acute lymphoblastic leukemia and B-cell lymphomas. However, relapse remains an issue in many cases. Such relapse can result from downregulation or loss of CD19 from the malignant cell population or expression of alternate isoforms. Consequently, there remains a need to target alternative B-cell antigens and diversify the spectrum of epitopes targeted within the same antigen. CD22 has been identified as a substitute target in cases of CD19-negative relapse. One anti-CD22 antibody—clone m971—targets a membrane-proximal epitope of CD22 and has been widely validated and used in the clinic. Here, we have compared m971-CAR with a novel CAR derived from IS7, an antibody that targets a central epitope on CD22. The IS7-CAR has superior avidity and is active and specific against CD22-positive targets, including B-acute lymphoblastic leukemia patient-derived xenograft samples. Side-by-side comparisons indicated that while IS7-CAR killed less rapidly than m971-CAR *in vitro*, it remains efficient in controlling lymphoma xenograft models *in vivo*. Thus, IS7-CAR presents a potential alternative candidate for the treatment of refractory B-cell malignancies.

B-cell acute lymphoblastic leukemia (B-ALL) frequently occurs as a pediatric malignancy (1, 2) but is also observed in adults (3). Conventional frontline treatment options are numerous; however, relapsed or refractory disease is common and the prognosis in such cases is dismal (1, 4, 5). Chimeric antigen receptor (CAR) T-cell therapy targeting CD19 has provided an additional option for B-ALL and other B-cell malignancies (6, 7, 8), yet relapse remains a significant challenge, occurring in 30 to 60% of the cases (9, 10). These may result from limited persistence of CAR T cells (11) or emergence of low/negative CD19 subpopulations of leukemic cells (12, 13).

This has led to a growing interest in other B-lineage markers such as CD20 (14), CD22 (7, 15), and CD79b (16). Despite this increased repertoire, relapse remains a challenge, as tumor cells evade CAR-T cell–mediated elimination by alternative splicing (12) or downregulation of cell surface markers (4, 15). One approach is to target multiple antigens, such as CD19 and CD22, whether from a bispecific “tandem” CAR (*e.g.*, 5) or by sequential administration of CARs with different specificities (*e.g.*, (17, 18)), thereby restricting the avenues of tumor escape. Development of such approaches is ongoing (5, 19). Meanwhile, the search continues for alternative targets and alternative epitopes on those targets.

For CD22, antibodies targeting different epitopes have been identified, with numerous CARs having been developed from these (18, 20, 21, 22, 23, 24). Several such CD22-directed CARs have entered clinical trials (15, 18, 22). However, the issues of refractory cases and relapse remain. It is anticipated that the development of CARs with different characteristics may help overcome these challenges. Early work with an anti-CD22 CAR (21) suggested that distal epitopes were less prone to generate efficient response in adoptive cell therapy, supporting the development of the m971-based CAR construct; this single chain variable fragment (scFv) was isolated from a human phage display library (25) and found to be bound to membrane-proximal domains 5 to 7 of the CD22 molecule (Fig. 1*A*, left). This is a unique feature among anti-CD22 antibodies, and the efficiency of this m971-based CAR has been attributed to an increased stability of the antibody–antigen complex (26). Recent clinical data also suggest that tonic signaling of the m971-CAR had a positive impact on T cell persistence in the patient cohort (27). Thus, it is still unclear to what extent epitope position influences the CAR T-cell therapeutic outcome.

**Figure 1:**
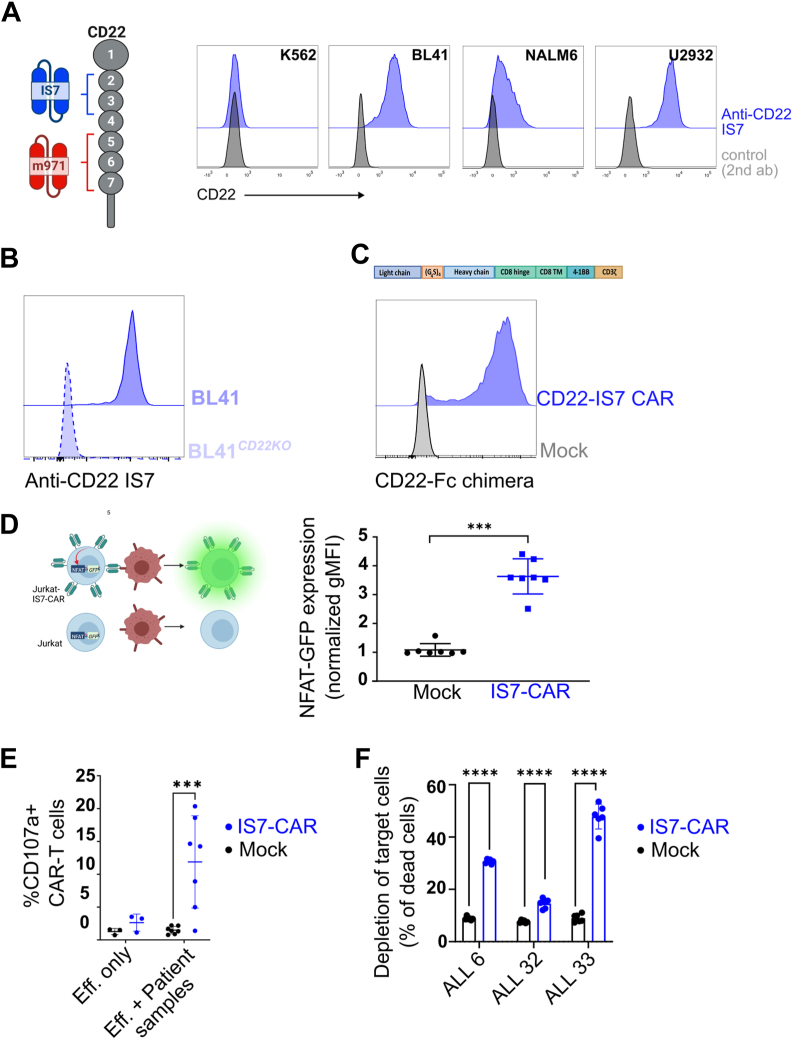
**Generation and design of IS7-CAR.***A*, *left*: representation of the CD22 domains and the position of the epitopes recognized by IS7 and m971 scFv. *Right*: indicated B cell lines were blocked with antihuman Fc, then labeled with a commercial IS7-APC antibody. K562 cells were used as a negative control. Background staining was detected using anti-mouse Fc immunoglobulin G-APC (isotype). *B*, CD22 CRISPR-KO BL41 cells were labeled with IS7-APC, and the signal was compared with one of the intact BL41 cells by flow cytometry. *C*, *upper panel*, design of the IS7-CAR construct using a second-generation format. *Lower panel*, representative flow data of Jurkat76 cells mock- or IS7-CAR–transduced using retrovirus and tested 3 days later for their ability to bind to a CD22-Fc chimera, which was in turn detected using an anti-Fc secondary antibody. *D*, *left*, description of the Jurkat76 reporter system (J76-NFAT-GFP) in which NFAT controls GFP production upon T-cell stimulation. *Right*, J76-NFAT-GFP were transduced with the IS7-CAR, or mock transduced, and cocultured with or without BL41 cells. GFP expression was measured by flow cytometry and plotted as the geometric mean fluorescent intensity (gMFI). In each group, GFP expression in the presence of BL41 cells was normalized to effector-only controls (mean ± SD, n = 7, unpaired *t* test, ∗∗∗*p* < 0.001). *E*, IS7-CAR or mock-transduced T cells were cocultured with B-ALL PDX samples. Coculture was at E:T = 2:1 for 5 h, in the presence of anti-CD107a antibody. The percentage of reactive T cells (CD107a degranulation) was measured by flow cytometry (mean ± SD, n = 7 (n = 3 for effector-only control). Two-way ANOVA with Šídák's multiple comparisons test, ∗∗∗*p* < 0.001). *F*, IS7-CAR T cells were cocultured with CFSE-labeled cells from three different B-ALL patient samples, for 4 h (E:T = from 2:1 to 10:1, pooled). Samples were then stained with 7AAD, and depletion (% of 7AAD+, CFSE+ cells) was assessed by flow cytometry (mean ± SD, n = 6, two-way ANOVA with Šídák's multiple comparisons test, ∗∗∗∗*p* < 0.0001). CAR, chimeric antigen receptor; B-ALL,B-cell acute lymphoblastic leukemia; PDX,patient-derived xenograft; scFv, single chain variable fragment; CFSE, carboxyfluoroscein succinimidyl ester.

We have identified the coding sequence of the anti-CD22 antibody–producing hybridoma clone IS7, which was previously shown to bind to domains 2 and 3 of CD22. These domains are distal from the cell surface, but not at the end (C terminus) of the CD22 molecule (20). To our knowledge, this targeting site is unique when compared to other anti-CD22 antibodies (20, 28). Recent anti-CD22 CARs have demonstrated that targeting of alternate CD22 epitopes can be effective (23, 24). However, these were not compared directly against the “gold standard” m971-derived CAR.

In the present study, we have developed an IS7-CAR and demonstrated that it is efficient, specific, and able to stimulate T cells. We then tested the hypothesis that targeting a proximal epitope leads to a full anti-CD22 response. To this end, we compared IS7 directly against the m971-scFv in different formats. The binding data showed an advantage for IS7 in soluble formats. However, the bispecific T-cell engager (BiTE) and CAR-format functional data, *in vitro* and *in vivo*, were comparable or sometimes to the advantage of m971. We concluded that scFv analysis was a poor predictor of CAR functionality in this case. Nevertheless, the IS7-CAR remains an attractive complementary product to treat B-cell malignancies.

## Results

### Validation of IS7 antibody as a functional CAR targeting CD22

The IS7 antibody was isolated 30 years ago and identified as CD22-specific (20). We confirmed this by labeling of CD22+ cell lines and a CD22 negative cell line, K562 (Fig. 1*A*, right). We further checked IS7 specificity for CD22 using a lymphoma cell line, BL41, with CD22 knocked out and detected no signal when stained with IS7 antibody (Fig. 1*B*). We identified the IS7 coding sequence and designed a scFv (Figs. 1*C* and S1*A*), which was subcloned into a second-generation CAR backbone composed of a CD8 hinge, a CD8 transmembrane domain, and a 4-1BB-CD3z signaling tail (29). J76 cells bearing an NFAT-GFP reporter construct, J76-NFAT-GFP, were transduced with IS7-CAR and labeled with a CD22-Fc chimera. As shown in Figure 1*C*, the CAR construct was highly expressed and able to bind its target (CD22-Fc chimera). We then tested the functionality of the IS7-CAR construct in an NFAT-GFP reporter system (30) (Fig. 1*D* left). Mock- or IS7-CAR-J76-NFAT-GFP were cocultured with CD22+ BL41 cells overnight and GFP was detected only in the presence of the CAR (Fig. 1*D* right), suggesting that the IS7 antibody can be converted to an efficient scFv, resulting in a specific CAR construct.

We then evaluated IS7-CAR efficiency against B-ALL isolated from patient samples. We transduced primary T cells with IS7-CAR (nontransduced cells included as “mock” control) and cocultured them with patient samples to detect CAR-dependent T-cell activation. We observed that IS7-CAR T cells were strongly reactive against patient samples, as detected by increase of the degranulation marker CD107a (Fig. 1*E*). In addition, we directly assessed direct tumor cell killing by 7AAD-staining (Fig. 1*F*). An important issue in CAR T-cell development is the possibility of toxicity, resulting from either nonspecific binding or on-target off-tumor activity. Accordingly, we tested the IS7-CAR, m971, and anti-CD19 FMC63-CAR T cells against healthy, autologous bone marrow (BM) cells to evaluate toxicity against hematopoietic stem cells, and we found no significant differences compared to the mock for the CD22CAR constructs (Fig. S1*B*). These data indicate that IS7-CAR can efficiently redirect T cells against CD22-positive cancer targets, including primary samples, and that the construct is safe.

### Comparison of scFv targeting different CD22 epitopes

**Figure 2:**
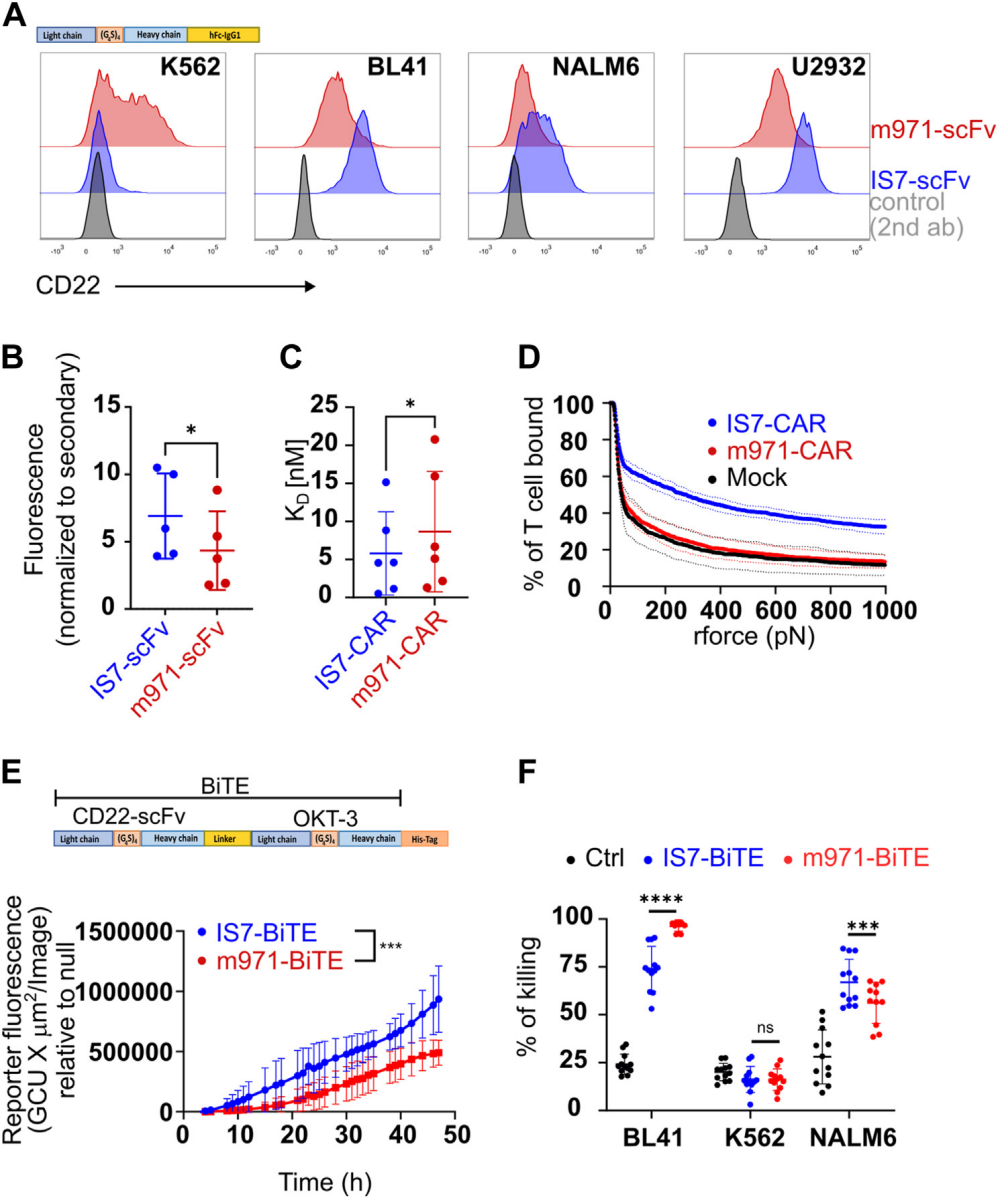
**Comparison of m971- and IS7-scFv using different formats.***A*, an erythroid/myelogenous leukemic cell line (K562), two B-cell lymphoma cell lines (BL41 and U2932), one B-ALL cell line (NALM6). *B*, B-ALL patient samples were labeled with IS7 and m971 soluble scFvs, then incubated with an anti-Fc secondary antibody. Geometric MFI (gMFI) was normalized to the Fc-only controls. (mean ± SD, paired two-tailed *t* test, n = 5. ∗*p* < 0.05). *C*, dissociation assay; IS7 and m971-CAR T cells were labeled with a dilution series of CD22-Fc chimeras. Secondary labeling with anti-Fc was performed at a constant concentration. (median ± interquartile range, Wilcoxon matched-pairs signed rank test, n = 6, ∗*p* < 0.05). *D*, in the z-Movi assay, CAR-transduced T cells were incubated with bound target (BL41) cells and subjected to increasing pressure until dissociation (two replicates from a single donor). *E*, Jurkat E6-NFAT-GFP reporter cells were cocultured with BL41 target cells (E:T = 1:1), in the presence of equal amounts of IS7- or m971-BiTEs (based upon His-tag labeling intensity on the Western blots, Fig. S1*C*). These were cultured in an IncuCyte apparatus, and the resulting reporter signal (GFP) was recorded over time. The signal was normalized against null controls (*i.e.*, HEK supernatants without BiTEs). *F*, IS7- and m971-BiTE were cocultured with activated T cells and the indicated CD22+ targets for 8 h at an E:T ratio = 1:1. BiTE-containing supernatant was standardized to 50% of the highest concentration recorded (Fig. S1*D*), then diluted 10-fold. (paired *t* test, mean ± SD, n = 4 donors in triplicate). CAR, chimeric antigen receptor; B-ALL,B-cell acute lymphoblastic leukemia; BiTE,bispecific T-cell engager; scFv, single chain variable fragment.

It was previously suggested that epitope position on CD22 was essential to generate an efficient anti-CD22 CAR construct (21, 26), as for the m971 construct. We therefore compared IS7 to m971 in different formats; scFv, CAR, and BiTE. We first constructed soluble forms of both scFvs, incorporating an Fc-tail, and used them to assess binding against CD22-positive target cells (Fig. 2*A*). In each case, labeling with the IS7 signal was higher. A background signal was observed with the negative cell line K562 using m971 scFv and was confirmed in strains obtained from different labs (not shown). Interestingly, m971 in CAR or BiTE format was not able to cause K562 killing (see below). These soluble scFv were used to label patient samples and we observed that the IS7-scFv labeled them with a higher intensity (Fig. 2*B*), suggesting that the epitope position did not alter binding efficiency. We then evaluated the binding of each CAR construct to its target by comparing their equilibrium dissociation constant (K_D_) (Fig. 2*C*). To this end, we loaded transduced J76-CAR cells with 10-fold dilutions of CD22-Fc chimera, decreasing from saturation, which were then labeled with anti-Fc and analyzed by flow cytometry. By assuming that our method follows sequential monovalent binding on each molecule, our observations point to a slightly, yet significantly, lower K_D_ of IS7-CAR than m971-CAR (Figs. 2*C* and S1*D*), suggesting that the latter has a lower functional avidity. These analyses were corroborated by a direct calculation of the applied force to separate CAR T cell from binding to its target using a z-Movi device. Here, we detected that the avidity of the IS7CAR design was markedly higher than that of m971 (Fig. 2*D*). Surprisingly, by this measure, the avidity of the m971-CAR was only a little higher than for mock T cells. From these data, we can conclude that IS7-scFv has at least similar, if not superior, binding properties and specificity to m971-scFv. Thus, the position of the epitope on CD22 protein evidently does not affect the interaction between the target antigen and antigen-binding domains, in whichever format. The scFv in the context of a CAR molecule loses some freedom to interact with targets due to the partial rigidity of the protein design. Thus, to further exclude any CAR design–related interferences in the binding and T cell activation, we produced BiTE constructs and tested their efficiency. It appeared that the m971-BiTE was more efficiently produced than IS7-BiTE (Fig. S1*C*). We therefore adjusted the concentration for the cellular assays. We used a Jurkat-NFAT-GFP reporter system, where the T cell line was cocultured with BL41 cells (E:T = 1:1) and the indicated BiTE construct. The GFP signal was quantified using an IncuCyte device. We observed that the IS7-BiTE construct induced a stronger reporter response (GFP signal) than did the m971-BiTE (Fig. 2*E*). Subsequently the BiTE constructs were tested with primary T cells and used in a bioluminescent-based killing assay. Activated T cells from four different donors were cocultured with the indicated target (E:T = 10:1) for 8 h, with or without BiTEs, and the target killing was quantified (Fig. 2*F*). Although both constructs efficiently killed the target cells, the m971-BiTE appeared more efficient than the IS7-BiTE with BL41 cells, which express more CD22, whereas NALM6 were more sensitive to IS7-BiTE (Fig. 2*F*). From these data, we concluded that although IS7-scFv bound more strongly to CD22 than m971-scFv, at the functional level in the BiTE format, both constructs were influenced by antigen density, rather than the avidity of the scFv.

### *In vitro* and *in vivo* comparison of CD22 CARs

**Figure 3:**
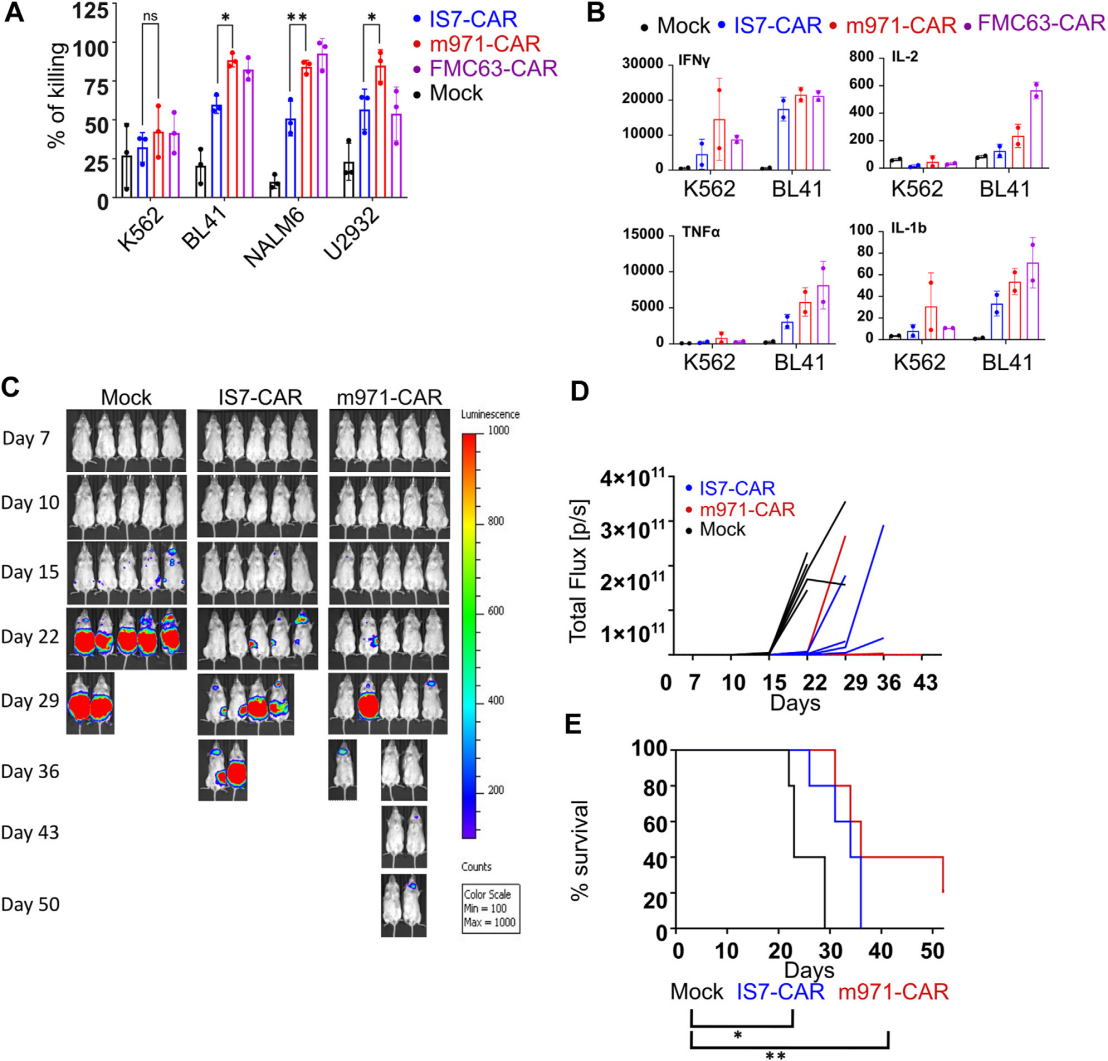
**Comparison of IS7- with m971-CAR *in vitro* and *in vivo*.***A*, bioluminescent-based killing assay of CAR-transduced T cells cocultured with the indicated targets (K562 is the CD22-negative control) for 12 h, at an E:T ratio of 10:1 (mean ± SD, two-way ANOVAs with Tukey’s multiple comparisons correction, n = 3 donors, ∗*p* < 0.032, ∗∗*p* < 0.0021); *p*-values are only shown for IS7-CAR *versus* m971-CAR. *B*, IS7- and m971-CAR T cells were cocultured with CD22+ (BL41) or CD22- (K562) target cells (E:T = 1:2) for 24 h. FMC63 (anti-CD19) CAR T cells were included for comparison. Supernatants were collected and analyzed by Bio-Plex assay. Data for the indicated cytokines are shown in ng/ml, n = 2 donors. Scale is relative, with values normalized to the K562 mock sample (summary data, n = 2). *C*, NSG (NOD.Cg-*Prkdc*^*scid*^*Il2rg*^*tm1Wjl*^*/SzJ*) mice were engrafted (i.v.) with 1 × 10^6^ luciferin-expressing BL41 cells. They were treated with two doses of 10 × 10^6^ CAR T cells (i.v.). Shown is the tumor development in these mice weekly analyzed by IVIS and quantified (*D*). *E*, Kaplan–Meier curve showing survival of mice throughout the experiment (log-rank test, n = 5, ∗*p* < 0.05, ∗∗*p* < 0.01). CAR, chimeric antigen receptor; i.v., intravenously; IVIS, in vivo imaging system; NOD, nonobese diabetic; NSG, NOD scid gamma mouse; scid, severe combined immunodeficiency.

We first tested IS7- and m971-CAR T cell cytotoxicity against different targets by bioluminescent-based killing assay. CAR-transduced primary T cells from three different donors were cocultured with CD22+ cell lines; BL41, NALM6, and U2932 (Fig. 3*A*). CD22-negative K562 cells were used as a negative control. We also included the anti-CD19 FMC63-CAR as a control (all of the target cells lines except K562 were positive for CD19, Fig. S2*A*). Initially, we tested each CAR design against target cells at an E:T ratio of 10:1. In agreement with CD22 staining and CAR reactivity, the IS7-CAR did not kill K562 cells but exhibited strong and specific killing of each of the CD22+ cell lines. However, this activity was statistically lower than the one observed with m971-CAR T cells (Fig. 3*A*). A similar trend was observed when we ran the experiment at different E:T ratios using BL41 cells (Fig. S2*B*). Here, K562 cells were used to exclude the possibility that m971-CAR was unspecific in our hands. These data demonstrate that IS7-CAR was specific but slightly less efficient than m971-CAR in the CAR format. These data were paralleled by the cytokine release capacity of the CD22-CAR T cells. In this experiment, T cells were cocultured with BL41 or K562 cells, and the supernatant was collected for the analysis of cytokines (Figs. 3*B* and S3). We used the anti-CD19 FMC63-CAR as a positive control. Here, the CD22 CARs appeared milder in their stimulatory activity than FMC63-CAR, and m971-CAR showed a greater interleukin-1 beta, interleukin-2, and tumor necrosis factor alpha release capacity than the IS7-CAR, whereas IFNγ levels were comparable. Thus, although a bit weaker than m971-CAR, IS7-CAR could efficiently stimulate a specific T-cell response against cancer cell lines. Next, we tested anti-CD22 CAR T cells in a BL41 xenograft model. Animals were treated with IS7- or m971-CAR T cells or mock-transduced T cells. Activity with both CAR designs was sufficient to control the tumors, up to 4 weeks, in contrast to the mock-treated mice (Fig. 3*C*). The latter had heavy tumor loads and all mice were euthanized by 29 days (Fig. 3, *D* and *E*). IS7- and m971-CAR T cell–treated mice met humane endpoints at around 30 days, with median survival of 26, 34, and 36 days for mock-, IS7-, and m971-treated groups, respectively (Fig. 3*E*). One mouse in the m971-treated group survived until the end of the study (day 52), but overall, both anti-CD22 CARs showed efficiency in partially controlling BL41 growth and led to an improved survival benefit over mock T cells, with—again—a tendency for the m971-CAR to be more effective. These results are in the same range as we previously reported with anti-CD19 CAR treatment (31). We also performed an *in vivo* experiment with the aggressive B-ALL cell line, NALM6 (Fig. S4). Here, both CARs delayed the expansion of tumor load and conferred a significant survival benefit, compared to mock. However, m971-CAR T cells controlled the tumor to a greater extent than IS7-CAR T cells (Fig. S4, A and B). This is reflected in the timing at which the three populations met humane endpoints (Fig. S4*C*). Taken together, these data show a significant anti-CD22 CAR control of different tumor models, with an advantage of m971-CAR over IS7-CAR. This suggests that factors other than functional avidity of the scFv govern CAR efficiency, both *in vitro* and *in vivo*.

## Discussion

The need for novel CARs that target alternative epitopes on validated cancer markers is clear; it has been shown that selective pressure of the CAR T cells upon tumors can lead to expression of antigen proteins lacking the recognized epitope (12). Herein, we have described an IS7-derived CAR, which is specific for an unexploited epitope on CD22. It was previously proposed that epitopes proximal to the cell surface were superior for CAR targeting than distal epitopes (21). However, to our knowledge, few subsequent studies have compared the membrane-proximal targeting m971-CAR with alternative CARs against CD22. One exception is the study of Pan *et al.* (22); however, the m971-CAR used therein appeared almost inactive. Since no indication about the heavy-light chain orientation of the m971-scFv was provided, we suspect that, based on our unpublished observations, their design has impacted the reported CAR function. Thus, their conclusions on the superiority of their novel “YK” CAR should perhaps be reassessed. In the present study, we compared both the binding (soluble scFv) and effector functions (BiTE/CAR format) of IS7 and m971 designs. Although IS7 more efficiently labeled CD22+ cells and stimulated NFAT reporter cells more strongly than m971, target cell killing—particularly in the CAR format—tended to be weaker with IS7. How can we explain this discrepancy?

In the case of NALM6 cell killing and the xenograft model, there was a dramatic contrast between the lower m971-scFv labeling and the effective m971-CAR killing. Yet, m971-CARs rapidly cleared all NALM6 cells, suggesting that the labeling did not reflect on what the CAR “saw.” One can speculate that CD22 expression by NALM6 cells is variable in time, thus the staining *per se* cannot predict how m971 scFv in a CAR—or to a lesser extent, in BiTE—format will behave. On the other hand, the superior binding kinetics observed with the IS7-scFv did not translate into higher functional capacities than m971. Thus, K_D_ determination might be a poor predictor of CAR functional avidity. We first speculated that m971 could detect a potential splice variant absent from the IS7 epitope but were not able to identify any such variants (data not shown). Another possibility could be a secondary modification that affects IS7 binding, but not m971. In this scenario, NALM6 might expose a CD22 molecule particularly well recognized by m971, but this characteristic was not detected in other cell lines or in primary samples. Thus, this seriously questions the validity of using NALM6 and cell lines in general, to select CARs. Finally, the epitope position might exclude CD45 from the immune synapse by reducing the distance available between the T cell and its target as recently proposed by Xiao and colleagues (32), which would support the “epitope position” model. At another level, it was suggested that an additional advantage of the m971-CAR lies in its tonic activity, that is, CAR activity in the absence of the target antigen recognition (27). Herein, we demonstrate similar target binding, and even killing, between IS7 and m971 outside the CAR context (*i.e.*, as soluble scFvs and BiTEs). Notably, this is the first demonstration, to our knowledge, that CD22 can be targeted by BiTEs. Thus, it appears that m971-CAR function is at least partly dependent upon nonspecific aspects of its design. Taken together, our data not only provide the validation of a novel attractive, candidate for anti-CD22–based therapy but also support more comparative studies where an existing product is already available. Whether IS7-CAR has a competitive potential against m971-CAR will only be supported by clinical testing.

In conclusion, our data demonstrate that targeting a distal epitope with an anti-CD22 CAR is possible and efficient. In addition, the BiTE format could also be exploited, and we here show that this is feasible. The standard CAR m971, which targets a membrane proximal domain, still appears slightly superior to IS7. We cannot fully explain the mechanism behind this discrepancy, but it does not exclude the future use of IS7-based therapeutics. This design adds another tool to the arsenal against CD22+ leukemias and may also provide an alternate, complementary approach to the treatment.

## Experimental procedures

### Patient samples

In Norway, peripheral blood mononuclear cells (PBMCs) were obtained from healthy donors under approval by the Regional Committee for Medical Research Ethics (REC approval no: 2019/121). Patient samples were obtained in accordance with the Declaration of Helsinki. In strict adherence with the approval of the Bioethics Committee of the Medical University of Warsaw and the Medical University of Lodz (KB/44/2015, RNN/51/19/KE) as well as with the patients’ written consent, primary blasts were isolated as described in (33). The primary material was then used for the generation of patient-derived xenografts (PDXs) in mice, in accordance with Ethics Committee of the Warsaw University and the Warsaw University of Life Sciences (287/2016, WAW2/095/2019) as described previously (33, 34). Successfully propagated PDX samples were used *ex vivo* for CD22 staining and for the anti-CD22 CAR-T killing assays.

Tumor biopsies were obtained from patients with follicular lymphoma (n = 4), diffuse large B-cell lymphoma (n = 16), and mantle cell lymphoma (n = 10) at the Norwegian Radium Hospital. Tonsils were obtained from patients (n = 8) undergoing tonsillectomy at Agroklinikken. Samples were processed to single-cell suspensions by mincing and cryopreserved in liquid nitrogen.

### Hybridoma sequencing and DNA constructs

Hybridomas of the IS7 clone were pelleted, the supernatant was decanted, and then frozen at −20 °C. They were later thawed, and the mRNA was collected for 5′ RACE, for sequencing of the heavy and light chains, as described previously (29). These were synthesized as a codon-optimized scFv (light chain–(G_4_S)_4_–heavy chain) and subcloned into a CAR backbone containing CD8-derived hinge and transmembrane domains, a 4-1BB costimulatory domain, and a CD3ζ intracellular domain, as previously described (35). The anti-CD19 scFv derived from FMC63 (a kind gift from Martin Pule, UCL Cancer Institute) was incorporated into our CAR backbone. The sequence of the m971 heavy and light chains was obtained from European patent EP2912061 and designed as a compatible scFv to be subcloned into the same CAR backbone.

Soluble scFvs were generated by cloning each scFv sequence into a pFUSE-hIgG-Fc plasmid (Invivogen). For generating BiTEs, scFv sequences were cloned into a plasmid based on the publicly available sequence of Blinatumomab (https://go.drugbank.com/drugs/DB09052).

To generate luciferase-expressing cell lines for bioluminescent and *in vivo* assays, we adapted a construct incorporating the firefly luciferase-GFP (a kind gift from Rainer Loew, BioNTech IMFS) (36) into the retroviral vector pMP71 (37). Cell lines were transduced, and stably expressing cells were sorted by fluorescence-activated cell sorting.

The NFAT-GFP construct for reporter assays (pSIRV-NFAT-eGFP) was a gift from Peter Steinberger (Addgene plasmid # 118031) (38).

For CRISPR KOs, we used MSCV_Cas9_puro, a gift from Christopher Vakoc (Addgene plasmid # 65655) (39), followed by puromycin selection to generate and maintain a stable line. These cells were subsequently electroporated with a guide RNA, targeting the proximal domain of CD22 (23). Knockout was confirmed by flow cytometry, following labeling for CD22 (clone HIB22). Negative cells were sorted by fluorescence-activated cell sorting, then expanded.

### Cell culture conditions

All *in vitro* culture was at 37 °C, with 5% CO_2_, in a humid environment. Most cell lines were cultured in RPMI (Biowest) with 10% FBS (Thermo Fisher Scientific) and 10 μg/ml gentamycin (Thermo Fisher Scientific). HEK-P cells were cultured in Dulbecco's modified Eagle's medium (Sigma) with 10% HyClone FBS (GE Healthcare Life Sciences) and 10 μg/ml gentamycin.

PBMCs were isolated from whole blood obtained from healthy donors under an approved institutional protocol. These cells were cultured in X-vivo 15 (Lonza), with 5% human serum (TCS Biosciences, Buckingham MK18 2LR) and IL-2 (Clinigen) at 100 U/ml. For activation, culture plates were precoated with 500 μl of anti-CD3 (functional grade OKT3, eBioscience) and anti-CD28 (functional grade CD28.6, eBioscience) antibodies, combined at 1 μg/ml each in PBS, for 2 h at room temperature. After incubation, this mixture was removed and PBMCs added at 10^6^ cells per ml, per well of a 24-well plate. After 2 to 3 days, cells were counted and ready for transduction.

### Production of retroviral vectors, transduction, and protein production

Production of retroviral particles and transduction of T cells were performed as described previously (37). Supernatants containing soluble scFvs and BiTEs were also produced by transfection of HEK-P cells, but without accessory plasmids, and the production media used was Iscove's Modified Dulbecco's Medium (Lonza) with 1% HyClone FBS at 32 °C for 48 h. Protein levels were quantitated by Western blotting, then supernatants were stored at 4 °C until use.

### Flow cytometry

Expression of the anti-CD22 CARs was detected by flow cytometry. Cells were labeled with a CD22-Fc chimera (Recombinant human Siglec-2/CD22 Fc chimera, R&D Systems, at 0.1 μg/100 μl), followed by incubation with an anti-Fc secondary antibody (antihuman immunoglobulin G Fc, BioLegend, 1/200 dilution of stock). Anti CD19-CAR expression was detected with a CD19 chimera (human CD19, Fc tag, Acro biosystems, at 0.3 μg/100 μl), followed by the same secondary antibody.

CD22 labeling was performed with various conjugated mAbs. These were HIB22 (BioLegend), S-HCL-1 (BD Biosciences), 4KB128, and IS7 (both Thermo Fisher Scientific).

### Coculture assays

For the reporter assays, Jurkat (J76) and Jurkat E6 cells were transduced with the NFAT-GFP reporter construct (38). These were cloned, and cells with a strong and specific response to stimulus (phorbol-myristate-acetate/ionomycin, not shown) were expanded to generate a J76- or JE6-NFAT-GFP clone. J76-NFAP-GFP were subsequently transduced with the CAR vectors, then cocultured with target cells at an Effector:Target (E:T) ratio of 1:2, overnight in 10% RPMI. CAR-mediated reactivity was assessed by GFP expression, as measured by flow cytometry. To assess the response of BiTEs on target cell, IS7- and m971-derived BiTEs were used in coculture with JE6-NFAT-GFP and BL41 (E:T = 1:1). The BiTE response was measured by GFP expression using the Live-Cell Analysis Instrument, IncuCyte (Sartorius).

Bioluminescence-based cytotoxicity assays were performed as previously described (40). CAR T cell functionality was assessed by degranulation detection. CAR-transduced T cells were cocultured with target cells (E:T = 1:2) for 5 h, in the presence of anti-CD107a, GolgiStop, and GolgiPlug (all BD Bioscience) at the recommended concentrations. Degranulation was assessed by detecting CD107a labeling with flow cytometry. Effector and target cells were distinguished by GFP expression where present, by prelabeling with CellTrace Violet, as per manufacturer’s methods (Thermo Fisher Scientific), or by labeling effectors for CD3 (BD Biosciences).

For analysis of cytotoxicity by the depletion of CD22-positive cells (PDX samples or cell lines), target cells were labeled with carboxyfluoroscein succinimidyl ester, or CellTrace Violet, as per the manufacturer’s methods. At the end of coculture with CAR T cells, 7-AAD or propidium iodide was added as per the manufacturer’s recommendations (both Thermo Fisher Scientific), and then samples were analyzed by flow cytometry.

For examination of cytokine secretion, CAR T cells were cocultured with target cells (E:T = 1:2) overnight in serum-free RPMI or X-vivo 15. After brief centrifugation (500*g* for 5 min), the supernatant was removed and immediately frozen at −80 °C until use. Samples were prepared as per the manufacturer’s methods (Bio-Plex Pro Human Cytokine Group I panel, 17-plex kit, Bio-Rad Laboratories).

### CFU assays

BM cells were obtained from healthy donors under approval by the Regional Committee for Medical Research Ethics. For colony-forming unit (CFU) assays, BM cells from healthy donors were cocultured for 6 h with autologous T cells transduced with CARs or mock-transduced. Cells were then transferred to semisolid methylcellulose–based culture medium. After 14 days, CFU-E, burst-forming unit-erythroid, CFU-granulocyte, monocyte, and CFU-granulocyte, erythrocyte, monocyte, megakaryocyte colonies were counted. Further details are available in the Supporting information.

### CD22 chimera binding

J76 cells were transduced with CAR vectors (IS7 or m971), then labeled with CD22-Fc chimera at 10-fold dilutions, from 0 to 100 μg/ml. They were subsequently stained with mouse anti-human immunoglobulin G Fc at saturation (1/20 dilution of stock). Median fluorescence intensity was determined for each sample and used for calculation of the equilibrium equation constant (K_D_) using the GraphPad Prism “one-site total binding equation” (GraphPad Software; https://www.graphpad.com/). This method was adapted from Jureczek *et al.* (41).

### z-Movi assay

We undertook CAR avidity assays, in accordance with the standard z-Movi protocol (Lumicks). Briefly, 20 to 30 μl of target cells (BL41 at 1 × 10^8^ cells/ml) were seeded on the chip and allowed to form a monolayer over 3 h. T cells were labeled with CellTrace Violet, and 20 to 30 μl (3 × 10^5^ cells total) was added to the chip. After 5 min incubation, increasing pressure was applied (0–1000 pN), and the proportion of CAR T cells remaining bound to the target cells was measured.

### *In vivo* experiments

Nonobese diabetic severe combined immunodeficiency gamma mouse (NOD.Cg-*Prkdc*^*scid*^ *Il2rg*^*tm1Wjl*^*/SzJ*) mice were bred in-house and maintained in pathogen-free conditions under an approved institutional animal care protocol. Six- to ten-week-old nonobese diabetic severe combined immunodeficiency gamma mouse mice were injected intravenously with 1 × 10^6^ luciferase-expressing BL41 or NALM6 cells in serum-free RPMI. After 2 days, mice were injected (intra-peritoneally) with 200 μl D-luciferin (20 mg/ml), and engraftment was confirmed by in vivo imaging system. Mice were allocated to each treatment group, such that each group had a similar representation of engraftment levels. On the same day, mice were injected intravenously with 10 × 10^6^ T cells from each treatment group, with mock-transduced T cells used as a control. Five days later, mice received a second injection of T cells in the same manner. In vivo imaging system analysis was repeated weekly, and the condition of the mice was assessed at least twice weekly. Humane endpoints were as outlined by the host facility. The *in vivo* studies were approved by the Norwegian Food Safety Authority.

### Statistical analysis

Comparisons between groups were assessed by various tests, as described in the accompanying Figure legends. Data were analyzed with Prism 9 software (GraphPad Software). A *p* value of <0.05 was considered statistically significant.

## Data availability

Data are to be shared upon request. Request should be sent to the corresponding authors: Sébastien Wälchli, sebastw@rr-research.no and Else Marit Inderberg, elsmar@rr-research.no.

## Supporting information

This article contains supporting information.

## Conflict of interest

The authors declare that they have no conflicts of interest with the content of this article.
