# Supplementary material for: Efficient chimeric antigen receptor targeting of a central epitope of CD22
Source: J Biol Chem. 2023 Jun 1;299(7):104883. doi: 10.1016/j.jbc.2023.104883 (PMC10331463; doi:10.1016/j.jbc.2023.104883)
Supplement: Supporting Figures S1–S4 [file mmc1.docx]

**Efficient chimeric antigen receptor targeting of a central epitope of CD22**

Nicholas Paul Casey^1^, Clara Helena Klee^1, *^, Anne Fåne^1, *^, Benjamin Caulier^1,2,3^, Agnieszka Graczyk-Jarzynka^4, 5^, Marta Krawczyk^4, 5^, Klaudyna Fidyt^4^, Sarah E. Josefsson^6^, Hakan Köksal^1^, Pierre Dillard^1^, Elzbieta Patkowska^4^, Malgorzata Firczuk^4, 5^, Erlend B. Smeland^6^, Magdalena Winiarska^4, 5^, June H. Myklebust^6^, Else Marit Inderberg^1, *^, Sébastien Wälchli^1, *^

(1) Translational Research Unit, Section of Cellular Therapy, Department of Oncology, Oslo University Hospital, Oslo, Norway

(2) Center for Cancer Cell Reprogramming (CanCell), Institute for Clinical Medicine, Faculty of Medicine, University of Oslo, Oslo, Norway

(3) Department of Molecular Cell Biology, Institute for Cancer Research, Oslo University Hospital, Oslo, Norway

(4) Department of Immunology, Medical University of Warsaw, Warsaw, Poland

(5) Laboratory of Immunology, Mossakowski Medical Research Institute, Polish Academy of Sciences, Warsaw, Poland

(6) Department of Cancer Immunology, Institute for Cancer Research, Oslo University Hospital, Oslo, Norway

*These authors contributed equally to this work

Corresponding authors: Sébastien Wälchli, [sebastw@rr-research.no](mailto:sebastw@rr-research.no) and Else Marit Inderberg, [elsmar@rr-research.no](mailto:elsmar@rr-research.no)

# Supporting Information - Methods

## CFU Assays

For Colony-Forming Unit (CFU) assays, CAR- or Mock-transduced T cells at day 11 post-transduction were co-cultured (E:T = 10:1) with autologous bone marrow cells for 6 hours. After incubation, cells were washed and adjusted to 2.5x10^5^ living BM cells/mL in a final volume of 300 μL. For this purpose, thawed BM cells were left to recover for 6 hours to estimate the true viability count (without effectors). The solution was then homogenized with 4mL of complete MethoCult™ medium (#H04434, STEMCELL Technologies, Vancouver, Canada), and 1.1 mL was transferred per well of a 6-well plate (in triplicate). After 10 to 14 days, Colony-forming unit-erythroid (CFU-E), Burst-forming unit-erythroid (BFU-E), colony-forming unit-granulocyte, macrophage (CFU-GM), and colony-forming unit-granulocyte, erythrocyte, macrophage, megakaryocyte (CFU-GEMM; mixed lineages) colonies were counted.

# Supporting information - Figures

#
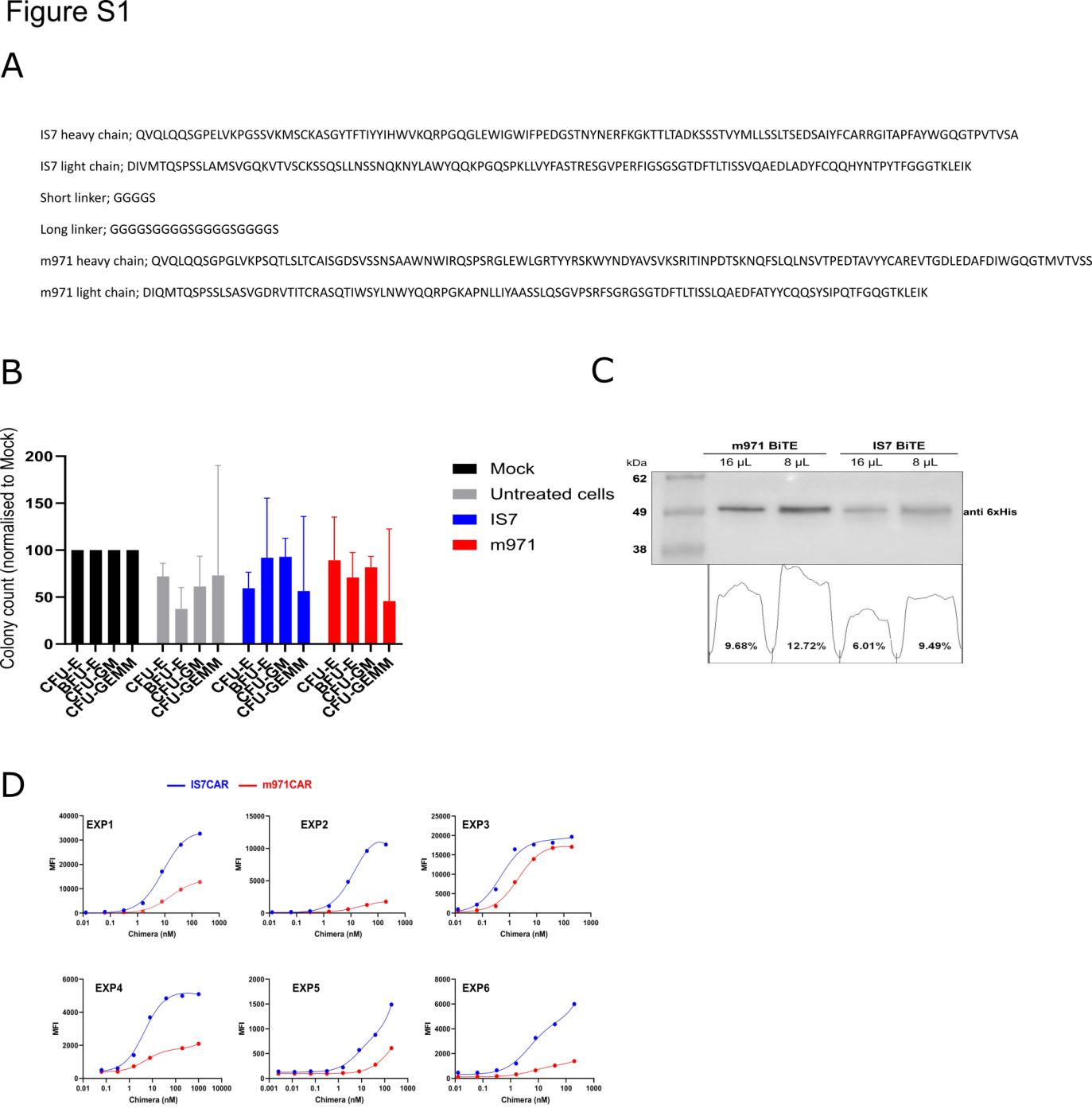


Figure S1. The IS7CAR is not toxic to haematopoietic cells.

A - The IS7 hybridomas were sequenced, and the heavy and light chain peptide sequences determined from these. The sequence of the short and long linkers is also indicated here, as are the heavy and light chain sequences of m971 (see text for source).

B – CAR T cells were co-cultured with CD34-enriched bone marrow cells, and the colony-forming units of each type were counted in triplicate. Data are mean of duplicate (N = 2) and error bars are S.D.

C– Supernatants from BiTE-producing HEK cells (volumes indicated) were Western blotted, and stained for the His-tag. Semi-quantitative assessment using Image-J allowed normalization of BiTE amounts in subsequent experiments.

D – Source data of the different experiments (EXP) performed to generate Figure 2C. Jurkat cells expressing the indicated CAR were incubated with 10-fold dilutions of CD22 chimera, labelled with anti-Fc and the presence of the chimera was detected as medium fluorescent intensity (MFI) by flow cytometry. The data were fitted in “One-site Total binding equation” in GraphPad and the K_D_ was extracted [nM]. N=6 separate experiments.


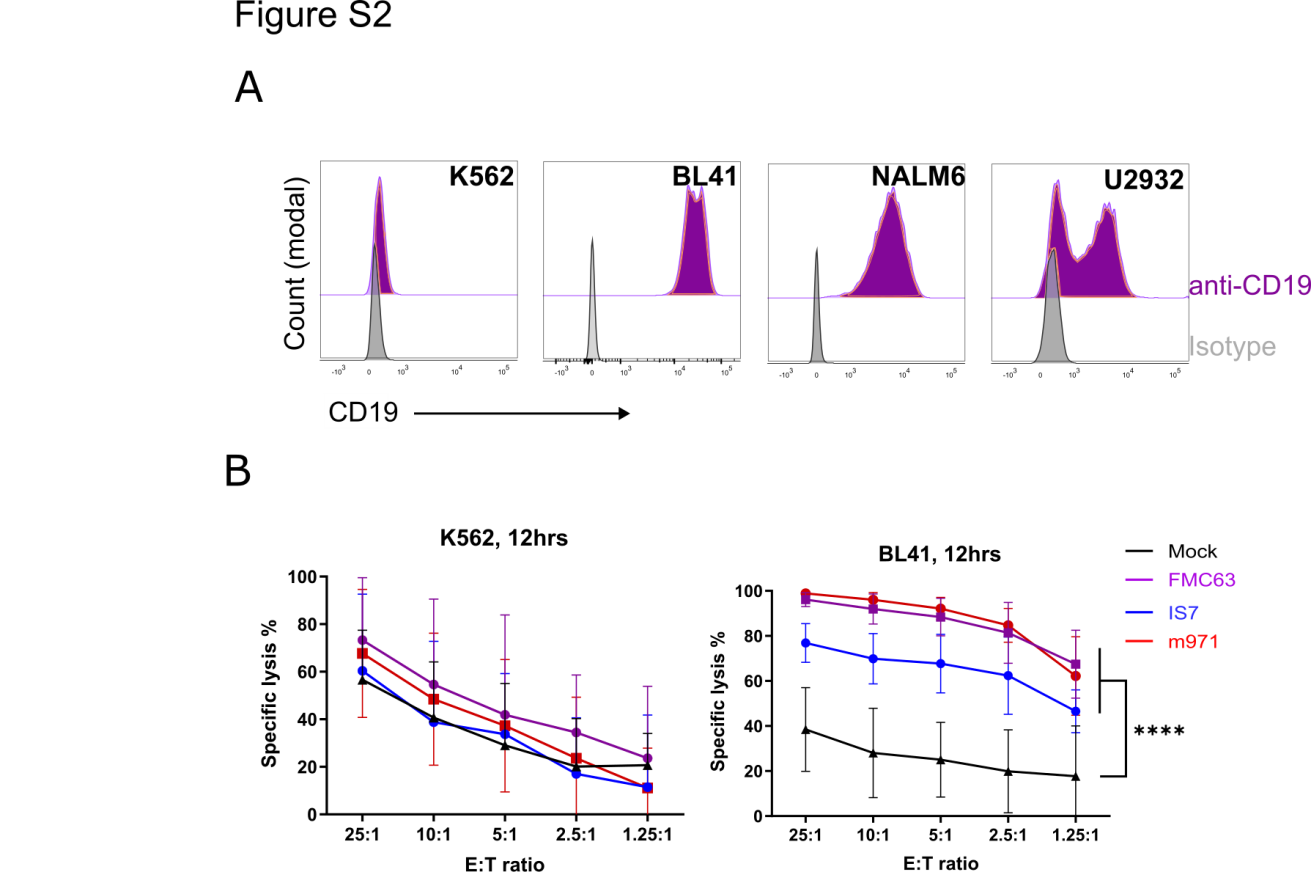


Figure S2. Both IS7- and m971-CAR efficiently kill CD22+ cell lines across several E:T ratios.

A – Staining of the cell lines used in the present study with anti-CD19 antibody

B – Bioluminescent-based killing assay co-cultures were repeated at a range of E:T ratios, from 25:1 to 1.25:1, as indicated. (Mean ± S.D., Two-way ANOVA with Dunnett’s multiple comparisons versus mock, n = 4. **** p < 0.0001).


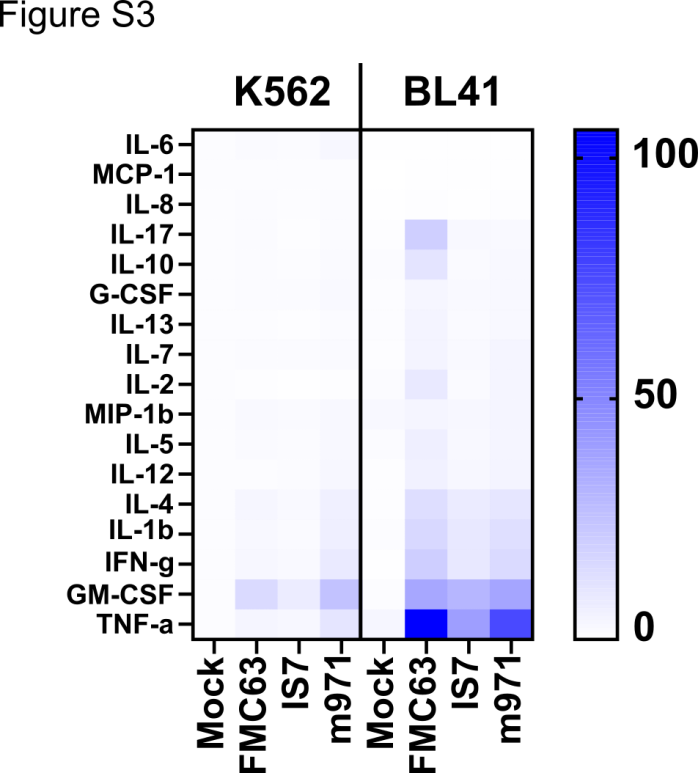


Figure S3. IS7- and m971 CAR T cells were co-cultured with CD22+ (BL41) or CD22- (K562) target cells (E:T = 1:2) for 24 hours. FMC63 (anti-CD19) CAR T cells were included for comparison. Supernatants were collected, and analyzed by BioPlex assay. Scale is relative, with values normalized to the K562 Mock sample (Summary data, n = 2).


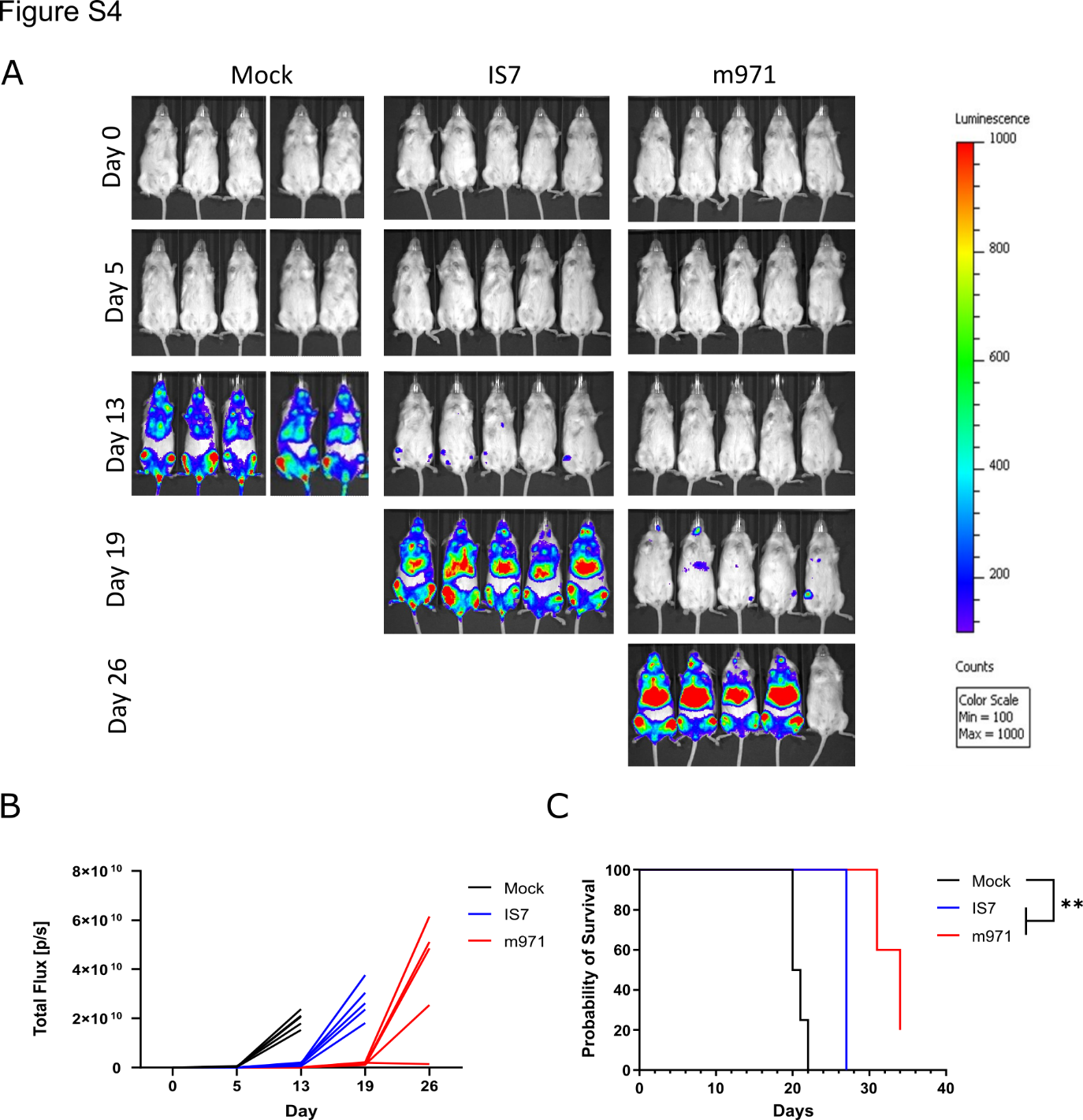


Figure S4. Both IS7- and m971-CAR reduce tumour growth and enhances survival in a NALM6 xenograft model.

A -NSG (NOD.Cg-*Prkdc^scid^ Il2rg^tm1Wjl^/SzJ*) mice were engrafted (i.v.) with 1x10^6^ luciferin-expressing NALM6 cells. They were treated with two doses of 10x10^6^ CAR T cells (i.v.). Tumor development in these mice was analyzed weekly by IVIS.

B – Quantification of tumor growth (total flux) for individual mice, after treatment with indicated CAR-T cells.

C – Kaplan-Meier curve showing survival of mice throughout the experiment (Log-rank test, n = 5, ** p < 0.01).
